# GLP-1 receptor agonist-associated tumor adverse events: A real-world study from 2004 to 2021 based on FAERS

**DOI:** 10.3389/fphar.2022.925377

**Published:** 2022-10-25

**Authors:** Zheng Yang, Yuhuan Lv, Meng Yu, Mei Mei, Linyu Xiang, Subei Zhao, Rong Li

**Affiliations:** Department of Endocrinology, The First Affiliated Hospital of Chongqing Medical University, Chongqing, China

**Keywords:** GLP-1RA, tumors, diabetes, FAERS, pharmacovigilance

## Abstract

**Background:** GLP-1 receptor agonists (GLP-1RA) have demonstrated cardiovascular benefits, but the relationship between GLP-1RA and tumors is controversial. Recently, clinical trials reported higher rates of malignancy with semaglutide than control group. As real-world evidence of GLP-1RA-associated tumor risk is very limited, we explored the association of GLP-1RA and all types of neoplasms by mining the FDA Adverse Event Reporting System (FAERS) database.

**Methods:** The FAERS data from the first quarter (Q1) of 2004 to the second quarter (Q2) of 2020 in the AERSMine were extracted to conduct disproportionality analysis, which was used by the proportional reporting ratio (PRR) to assess the relationship between GLP-1RA and all types of neoplasms. Then, the details of disproportionate GLP-1RA-associated tumor cases from Q1 2004 to Q2 2021 in the FAERS Public Dashboard were collected to analyze demographic characteristics.

**Results:** A total of 8718 GLP-1RA-associated tumors were reported. Excluding cases with pre-existing tumors, other glucose-lowering drugs, and other GLP-1RA-related adverse events, diabetes cases with GLP-1RA as the main suspected drug were selected. GLP-1RA did not cause a disproportionate increase in all tumor cases (PRR 0.83) at the SOC level, and there was also no increase in most types of tumors associated with GLP-1RA at the HLGT/HLT levels. Significant signals were detected between GLP-1RA and certain tumors, including thyroid cancers [medullary thyroid cancer (PRR 27.43) and papillary thyroid cancer (PRR 8.68)], pancreatic neoplasms malignant (PRR 9.86), and islet cell neoplasms and APUDoma NEC (PRR 2.86). The combination of GLP-1RA with dipeptidyl-peptidase IV inhibitors (DPP4i) perhaps caused the increased reporting rate in some tumors.

**Conclusion:** Our study provided new real-world evidence for oncology safety information of GLP-1RA. Given the wide use of GLP-1RA, clinicians should be well informed about important potential adverse events. Our pharmacovigilance analysis also prompted clinicians to raise concerns about potential tumor-related adverse effects when combining GLP-1RA with DPP4i.

## Introduction

Glucagon-like peptide-1 receptor agonists (GLP-1RA) are a group of important medications for patients with type 2 diabetes mellitus (T2DM). Accumulating evidence shows that GLP-1RA has established efficacy and safety profiles (Nauck et al., 2021). Due to the cardiovascular benefits, GLP-1RA or sodium-dependent glucose transporter 2 inhibitors (SGLT2is) are recommended for T2DM patients with established atherosclerotic cardiovascular disease (ASCVD) or high-risk indicators of ASCVD (American Diabetes Association, 2021). Given the wide use of GLP-1RA in T2DM, the relationship between GLP-1RA and tumors has always been a concern. Several meta-analyses indicated that GLP-1RA did not increase the risk of malignancy (Cao et al., 2019; Liu et al., 2019). However, Elashoff et al., analyzing the 2004–2009 US Food and Drug Administration (FDA) database, found that thyroid cancer and pancreatic cancer were more commonly reported in patients using exenatide than those using rosiglitazone (Elashoff et al., 2011).

Recently, the relationship between increasing types of tumors and GLP-1RA has attracted attention, including breast cancer (Hashimoto Takigami et al., 2021) and cholangiocarcinoma (Ueda et al., 2021). In 2020, a clinical trial in patients with nonalcoholic steatohepatitis (NASH) showed that neoplasms (including cysts and polyps) were reported in 15% (35/239) patients using semaglutide vs. 8% (6/80) with control treatment (Newsome et al., 2021). In 2021, STEP 3 (Wadden et al., 2021) and STEP 4 (Rubino et al., 2021) reported the incidence of malignancy in overweight or obese adults as 0.7% (3/407) and 1.1% (6/535) in the semaglutide group and 0.5% (1/204) and 0.4% (1/268) in the control group, respectively. In addition, Wang using the EHR database (Explorys; IBM Corporation, Armonk, New York, United States) reported that GLP-1RA was associated with a lower incident risk of prostate cancer [the adjusted odds ratio (aOR) 0.81], lung cancer (aOR 0.81), and colon cancer (aOR 0.85) and a higher incident risk of thyroid cancer (aOR 1.65) than metformin, and they also found similar association between these tumors and GLP-1RA in the FDA Adverse Event Reporting System (FAERS) database (Wang and Kim, 2022).

Due to the increasing use of GLP-1RA in T2DM, the potential impact of adverse effects deserves concern. The link between GLP-1RA and tumors is controversial, and the increasing types of GLP-1RA-associated tumor adverse events (AEs) have generated more attention. Therefore, the association between GLP-1RA and other types of tumors may remain undetected. Although meta-analyses suggested that GLP-1RA was not associated with tumor risk, meta-analyses have certain limitations. Subjects of the randomized controlled trial (RCT) are usually selected from relatively healthy individuals, patients with a history of MTC or pancreatitis may be excluded, RCT are not long enough to cause cancer AEs, and no RCT has tumor events as the primary outcome. Spontaneous reporting may be the best way to find rare AEs, and real-world evidence of GLP-1RA-associated tumor risk is currently limited. To address these queries and more fully understand the potential risk of all neoplasms related to GLP-1RA, we explored the association between GLP-1RA and tumors by mining the FAERS database.

## Materials and methods

### Data sources and procedures

The FAERS database contains AEs submitted to the FDA from the first quarter (Q1) of 2004 to the second quarter (Q2) of 2021. AERSMine, a web-based analyzing application, was designed to mine the FAERS data from 2004 Q1 to 2020 Q2 (Sarangdhar et al., 2016). AEs in the FAERS database are reported according to the MedDRA dictionary from system organ class (SOC), high-level group terms (HLGT), high-level terms (HLT), preferred terms (PT), and lowest level terms (LLT) (Brown et al., 1999).

First, we obtained the frequencies of GLP-1RA-associated neoplasm cases from AERSMine based on the search term developed by MedDRA (23.1), in order of SOC, HLGT, and HLT. The reporting rates of GLP-1RA-associated neoplasms were compared with different comparators in five models: 1) other drugs excluding GLP-1RA (non-GLP-1RA) without indication restrictions; 2) non-GLP-1RA when diabetes as an indication because diabetes itself increases the risk of cancers; 3) patients were excluded who already had tumors before GLP-1RA therapy, and GLP-1RA was reported as the “primary suspect” drug in all cases on the basis of model 2; 4) to rule out the effect of other glucose-lowering agents, and all cases combining other glucose-lowering drugs were excluded on the basis of model 3; and 5) GLP-1RA was not necessarily the primary suspect for tumors because there were many drug–AE pairs in one report; therefore, a sensitivity analysis was performed after excluding all cases with other AEs (retinal adverse events, acute kidney injury, hypoglycemia, nausea, vomiting, diarrhea, and pancreatitis) on the basis of model 4.

Then, we downloaded the FAERS files from 2004 Q1 to 2021 Q2 and collected the details of tumor cases with the lower limit of PRR invariably greater than 1 in five models. The following details were retrieved: the safety report ID, demographic characteristics, suspect and concomitant drugs, concomitant complications, outcomes, and reporting source.

### Statistical analysis

The proportional reporting ratio (PRR) is used to evaluate the generation of signals in the surveillance database, which is calculated as follows (Evans et al., 2001):

$$\frac{\left(\frac{\left[\text{number}\;\text{of}\;\text{neoplasms}\;\text{reports}\;\text{for}\;\text{GLP}-1\text{RA}\right]}{\left[\text{total}\;\text{number}\;\text{of}\;\text{reports}\;\text{for}\;\text{GLP}-1\text{RA}\right]}\right)}{\left(\frac{\left[\text{number}\;\text{of}\;\text{neoplasms}\;\text{reports}\;\text{for}\;\text{comparator}\;\text{drugs}\right]}{\left[\text{total}\;\text{number}\;\text{of}\;\text{reports}\;\text{for}\;\text{comparator}\;\text{drugs}\right]}\right)}.$$

A PRR of 1 indicates that GLP-1RA-associated neoplasms were reported as frequently as comparator-associated neoplasms. A PRR greater than 2 indicates that the reporting rate of GLP-1RA-associated neoplasms was more than twice the rate of comparator-associated neoplasms. *p* < 0.05 was considered statistically significant for reported disproportionality. We used SPSS 23 to analyze the data.

## Results

### Glucagon-like peptide-1 receptor agonist-related various neoplasms in system organ class, high-level group terms, and high-level terms

**FIGURE 1:**
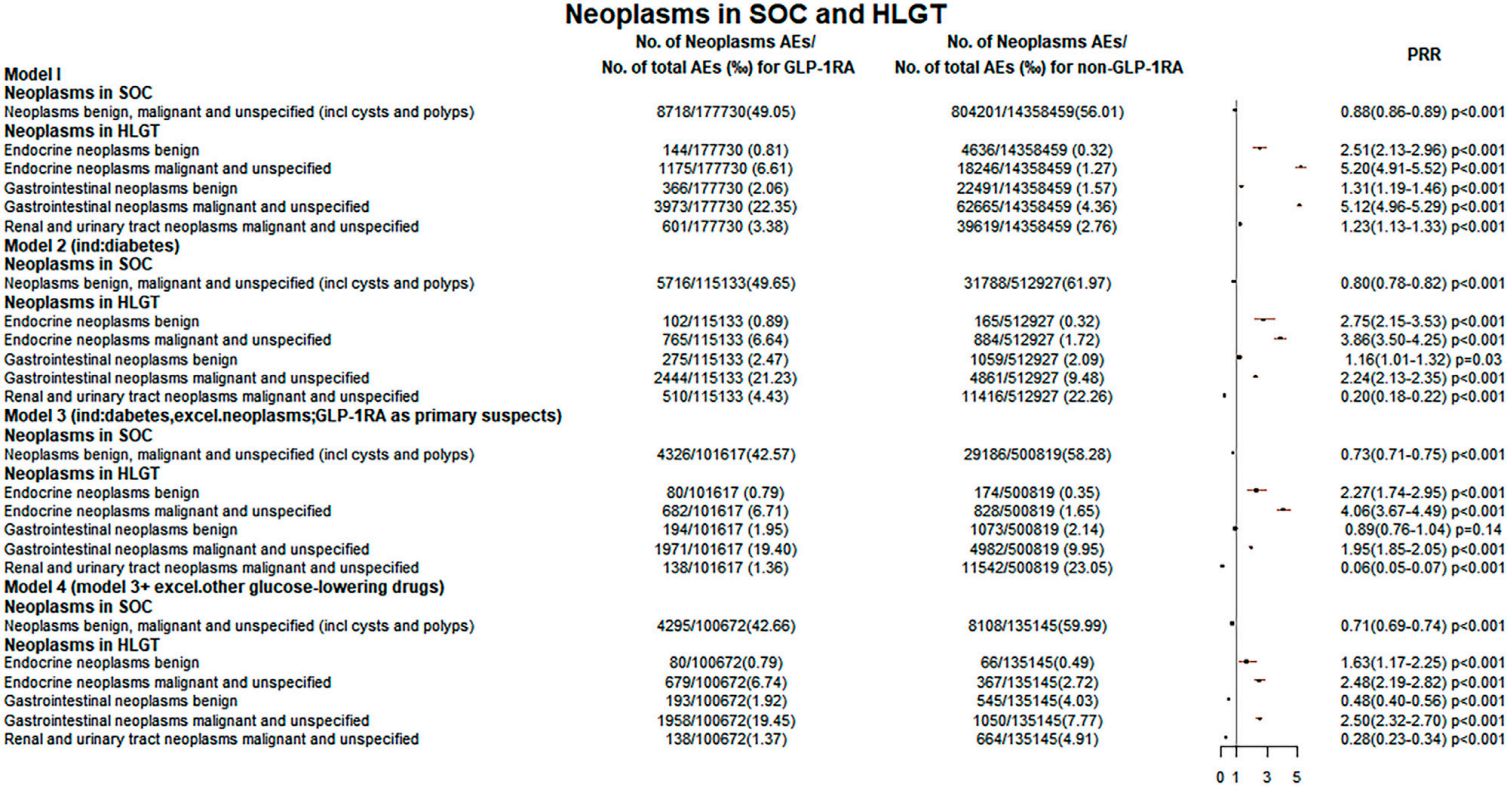
Neoplasm reporting rate at the SOC and HLGT levels. The reporting rates of GLP-1RA-associated neoplasms were compared with different comparators in four models: 1) other drugs excluding GLP-1RA (non-GLP-1RA) without indication restrictions; 2) non-GLP-1RA when diabetes as an indication; 3) non-GLP-1RA when the indication is limited to diabetes while excluding all tumors, and all cases with GLP-1RA as the “primary suspect” drug; and 4) all cases combining other glucose-lowering drugs were excluded on the basis of model 3 for comparison.

During 2004 Q1–2020 Q2, the FAERS files contained 8718 GLP-1RA-associated neoplasm reports. In five models, the reporting rates of tumors at the SOC level were lower in the GLP-1RA group than the non-GLP-1RA group (model 1: 49.1‰ vs. 56.0‰, PRR 0.88; model 2: 49.6‰ vs. 62.0‰, PRR 0.80; model 3: 42.6‰ vs. 58.3‰, PRR 0.73; model 4: 42.7‰ vs. 60.0‰, PRR 0.71; and model 5: 50.5‰ vs. 60.7‰, PRR 0.83). All these data are shown in Figure 1 Figure 5.

**FIGURE 2:**
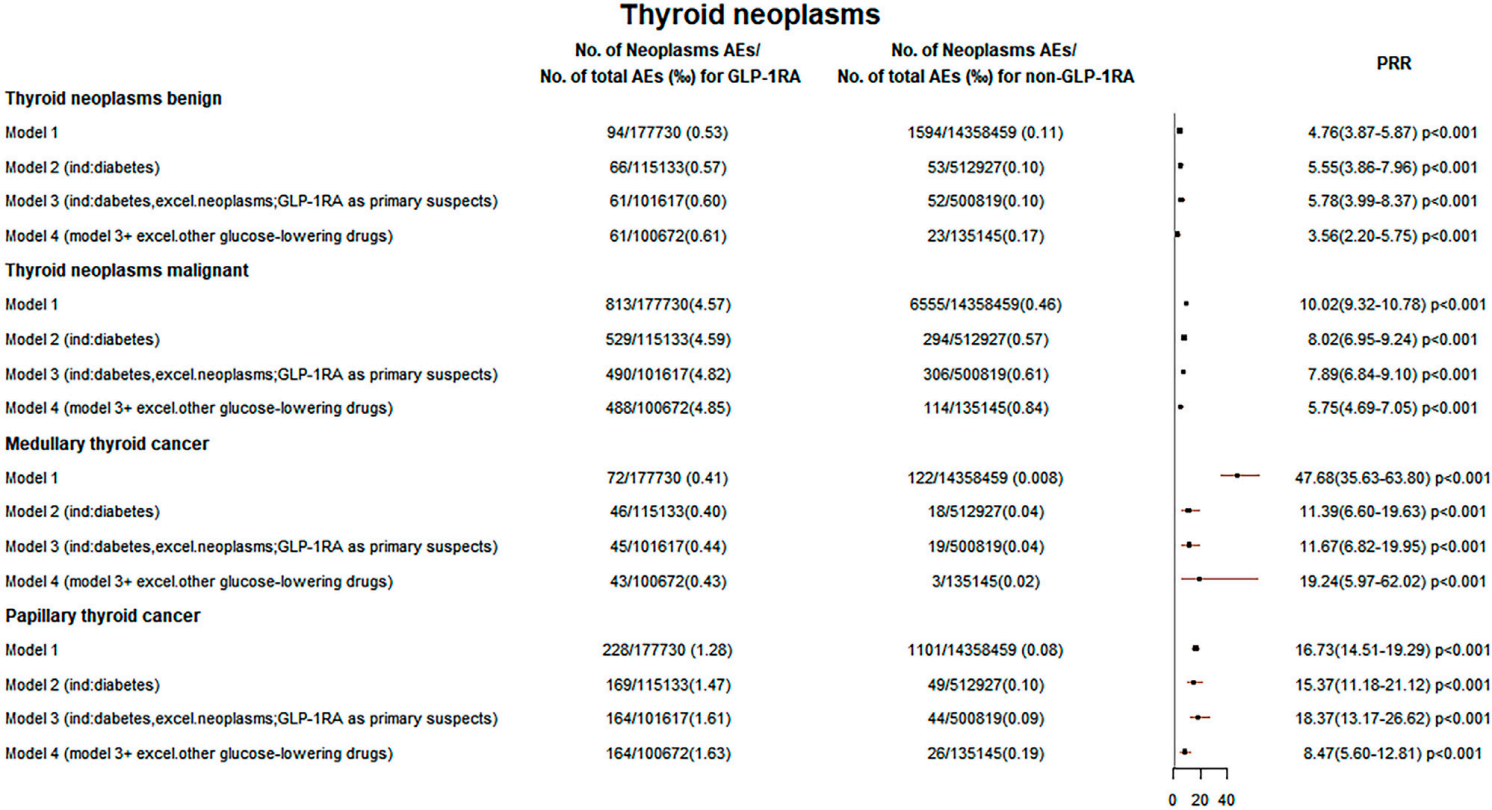
Thyroid neoplasm reporting rates. The reporting rates of GLP-1RA-associated neoplasms were compared with different comparators in four models: 1) other drugs excluding GLP-1RA (non-GLP-1RA) without indication restrictions; 2) non-GLP-1RA when diabetes as an indication; 3) non-GLP-1RA when the indication is limited to diabetes while excluding all tumors, and all cases with GLP-1RA as the “primary suspect” drug; and 4) all cases combining other glucose-lowering drugs were excluded on the basis of model 3 for comparison.

**FIGURE 3:**
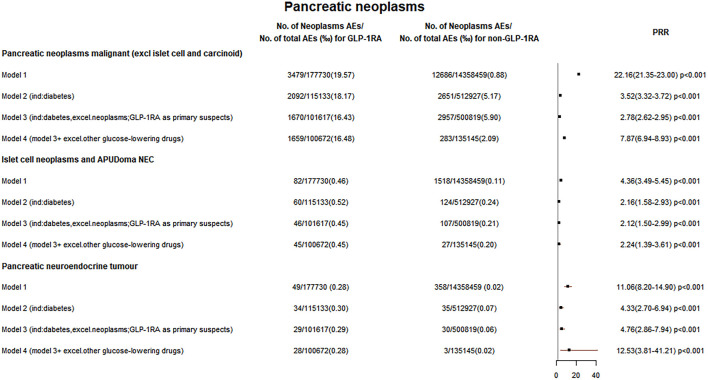
Pancreatic neoplasm reporting rates. The reporting rates of GLP-1RA-associated neoplasms were compared with different comparators in four models: 1) other drugs excluding GLP-1RA (non-GLP-1RA) without indication restrictions; 2) non-GLP-1RA when diabetes as an indication; 3) non-GLP-1RA when the indication is limited to diabetes while excluding all tumors, and all cases with GLP-1RA as the “primary suspect” drug; and 4) all cases combining other glucose-lowering drugs were excluded on the basis of model 3 for comparison.

In Supplementary Figures S1–S5, five models were used to analyze the relationship between GLP-1RA and all tumors at the HLGT level. Three groups of GLP-1RA-associated HLGT tumor reporting rates (endocrine neoplasms benign, endocrine neoplasms malignant and unspecified, gastrointestinal neoplasms malignant and unspecified) were higher than those of non-GLP-1RA, as shown in Figures 1, 5. Then, the PRR of all GLP-1RA-associated tumors at the HLT level (under the HLGT level) was calculated. The lower limit of PRR for the following HLT tumors was invariably greater than 1 in five models: thyroid neoplasms benign and malignant, pancreatic neoplasms malignant (excl islet cell and carcinoid), and islet cell neoplasms and APUDoma NEC (ICN&AN). All these data are shown in Figures 2, 3, and Figure 5.

### Glucagon-like peptide-1 receptor agonist-related thyroid neoplasms

In five models, the PRR for GLP-1RA-associated thyroid neoplasms benign was greater than 3 (PRR for models 1–5: 4.76, 5.55, 5.78, 3.56, and 4.03), and the PRR for thyroid neoplasms malignant was greater than 5 (PRR for models 1–5: 10.02, 8.02, 7.89, 5.75, and 6.89). All these data are shown in Figures 2, 5.

Then, we retrieved GLP-1RA-associated thyroid neoplasms benign (85 cases) and malignant reports (698 cases) from the FAERS public dashboard. Demographic analysis showed wide ranges of age (thyroid neoplasms benign: mean 58.9 ± 10.0 years and range 40–83 years; thyroid neoplasms malignant: mean 50.9 ± 16.2 years and range 20–83 years) and weight (thyroid neoplasms benign: mean 100.6 ± 22.1 kg and range 70–181 kg; thyroid neoplasms malignant: mean 102.7 ± 24.6 kg and range 46–191.8 kg). In thyroid neoplasms benign cases, the largest proportion was benign neoplasm of the thyroid gland (55.3%), followed by the thyroid cyst (24.7%) and thyroid adenoma (17.7%). In thyroid neoplasms malignant cases, papillary thyroid cancer (PTC) accounted for 28.9%, followed by medullary thyroid cancer (MTC) (11.0%). A total of 36 thyroid neoplasms malignant cases (3.9%) were fatal. These data are summarized in Supplementary Tables S1, S2.

### Glucagon-like peptide-1 receptor agonist-related pancreatic neoplasms

In five models, the PRR was greater than 2 for GLP-1RA-associated pancreatic neoplasms malignant (PRR for models 1–5: 22.16, 3.52, 2.78, 7.87, and 9.86) and ICN&AN (PRR for models 1–5: 4.36, 2.16, 2.12, 2.24, and 2.86). These data are shown in Figures 3, 5.

Then, the detailed data of pancreatic neoplasms malignant (2910 cases) and ICN&AN (78 cases) associated with GLP-1RA were obtained. The mean age was 64.1 ± 9.0 years (range 34–91 years) for pancreatic neoplasms malignant cases and 59.1 ± 9.3 years (range 32–84 years) for ICN&AN cases. The mean weight was 93.7 ± 24.6 kg (range 20.0–261.1 kg) for pancreatic neoplasms malignant cases and 103.5 ± 21.3 kg (range 62.6–159.2 kg) for ICN&AN cases. Among pancreatic neoplasms malignant reports, pancreatic carcinoma accounted for the majority (91.1%), followed by pancreatic carcinoma metastatic (20.1%). The vast majority of ICN&AN cases were pancreatic neuroendocrine tumors (85.9%), followed by pancreatic neuroendocrine tumor metastatic (24.4%). In outcome data, 1595 pancreatic neoplasms malignant (54.8%) and 14 ICN&AN cases (18%) were reported as deaths. All of these data are summarized in Supplementary Tables S3, S4.

### Glucagon-like peptide-1 receptor agonist-related other neoplasms

Additionally, the disproportionality of other GLP-1RA-associated tumor reporting rates was not observed. The upper limit of PRR for most GLP-1RA-associated tumors at the HGLT/HLT levels was robustly less than 1 by continuously excluding influencing factors in five models, including respiratory and mediastinal, breast, most male and female reproductive, bone and skin soft tissue, nervous system, ocular, and hematologic neoplasms. Similarly, the reporting rate of GLP-1RA-associated metastases did not show disproportionality. These data are shown in Supplementary Figures S1–S5.

## Discussion

By constantly excluding influencing factors in five models, the relationship of GLP-1RA with all tumors was explored in the FAERS database and an exhaustive pharmacovigilance analysis of tumor AEs was conducted at the SOC/HGLT/HLT levels. GLP-1RA-associated overall tumor at the SOC level and most types of tumors at the HGLT/HLT levels did not show a significant signal. However, disproportionality analyses showed that GLP-1RA is associated with thyroid neoplasms benign and malignant, pancreatic neoplasms malignant, and ICN&AN cases at the HLT level.

### Glucagon-like peptide-1 receptor agonist-related thyroid neoplasms

In our study, the increased reporting rate of GLP-1RA-associated thyroid tumors (thyroid neoplasms benign and malignant) in five models was observed. In thyroid neoplasms malignant cases, the most frequently combined hypoglycemic agent was metformin (194 cases, 71.1%). In thyroid neoplasm cases, the other most frequently combined drug was Synthroid (thyroid neoplasms benign: 8 cases, 19.5%; thyroid neoplasms malignant: 84 cases, 26.9%), which may indicate that these patients had hypothyroidism. Also, a meta-analysis reported that hypothyroidism was associated with a higher risk of thyroid cancer within the first 10 years of follow-up (Tran et al., 2020).

There may be several reasons for such a significant increase in the GLP-1RA-associated thyroid tumor reporting rate. First, the increased diagnosis rate of thyroid cancer may improve the reporting rate of AEs. The International Agency for Research on Cancer (IARC) surveyed thyroid cancer incidence trends across 25 countries and showed that the incidence of thyroid cancer, especially PTC has increased rapidly in recent years due to over-screening for the disease (Miranda-Filho et al., 2021). Second, since the FDA previously warned about the possible increased risk of MTC with GLP-1RA, this may prompt reporters to attribute more thyroid tumors including PTC to GLP-1RA. Then, strong attention to GLP-1RA-associated thyroid cancer may prompt more patients with GLP-1RA therapy to undergo thyroid ultrasound, thereby improving the detection of thyroid tumors and leading to an increase in thyroid tumor reports.

Figures 2, 5 show that the PRR for MTC and PTC was greater than 8 in five models. The predominance of females in thyroid tumors may be due to the higher risk of thyroid cancer in females (Rahbari et al., 2010). Supplementary Table S2 shows that MTC and PTC accounted for 11.0% and 28.9% of all thyroid neoplasms malignant cases, respectively. Data from the IARC during 1998–2012 showed that MTC and PTC accounted for 2.30% and 87.27% of all thyroid cancer types, respectively (Miranda-Filho et al., 2021). This may indicate an increased proportion of MTC in thyroid cancers in our study, and the increased GLP-1RA-associated MTC reporting rate also supports the FDA’s warning. The relationship between GLP-1RA and MTC remains a controversy. A study suggested that GLP-1 receptor (GLP-1R) localization exists in C cells of rodents and the activation of GLP-1R by GLP-1RA may promote C-cell proliferation and tumor formation, but for humans, a causal relationship between GLP-1RA and thyroid C-cell pathology has not been demonstrated (Bjerre Knudsen et al., 2010; Gier et al., 2012a).

The reason for the increased GLP-1RA-associated PTC reporting rate is unclear. There may be several reasons. Warnings that GLP-1RA increases the MTC risk may attribute PTC reports to GLP-1RA. Then, GLP-1R was also expressed in PTC cells, and GLP-1R exhibited immunoreactivity in 18 of 56 (32.1%) PTC cases (Jung and Kwon, 2014). A study showed that the expression of GLP-1R was more obvious in PTC cells than in normal thyroid cells, but GLP-1RA had no significant effect on PTC cell proliferation *in vitro* (He et al., 2017). The activation of GLP-1R by GLP-1RA may be potentially linked to the development of PTC. Therefore, the relationship between GLP-1RA and PTC may require long-term observation.

### Glucagon-like peptide-1 receptor agonist-related pancreatic neoplasms

First, the increased reporting rate of GLP-1RA-associated pancreatic neoplasms malignant was observed. The relationship between GLP-1RA and pancreatic cancer is still unclear. On the one hand, long-term use of GLP-1RA may be associated with an increased risk of pancreatitis (Gier et al., 2012a), which is an important risk factor for pancreatic cancer. GLP-1RA could promote pancreatic inflammation and increase serum lipase levels in rats (Nachnani et al., 2010). On the other hand, Gier et al. found that rats developed columnar cell atypia resembling low-grade pancreatic intraepithelial neoplasia which is considered a precursor to cancer after 12 weeks of exenatide treatment (Gier et al., 2012b). Butler et al. reported that GLP-1RA could cause pancreatic hyperplasia and dysplasia in brain-dead organ donors (Butler et al., 2013). However, a 2-year toxicity study showed that liraglutide did not increase pancreatic AEs in mice, rats, or monkeys (Nyborg et al., 2012). Subsequent studies also suggested that pancreatic histology was not altered by 18 weeks of GLP-1RA treatment in young mice (Cox et al., 2017).

Meanwhile, Figure 3 suggests that the reporting rate of GLP-1RA-related ICN&AN cases was increased. Supplementary Table S4 Pancreatic neuroendocrine tumors (PNETs) (85.9%) accounted for the majority of ICN&AN. PNETs may arise from pluripotent stem cells of the pancreatic duct/pancreatic system (Schimmack et al., 2011; Vortmeyer et al., 2004). Also, GLP-1RA can promote pancreatic hyperplasia and GLP-1R expression in the pancreatic duct (Nyborg et al., 2012). Meanwhile, a study found that 23 of 50 patients with PNETs had positive GLP-1R expression, and 8 of 11 metastatic PNETs had GLP-1R-positive metastasis sites (Cases et al., 2014).

**FIGURE 4:**
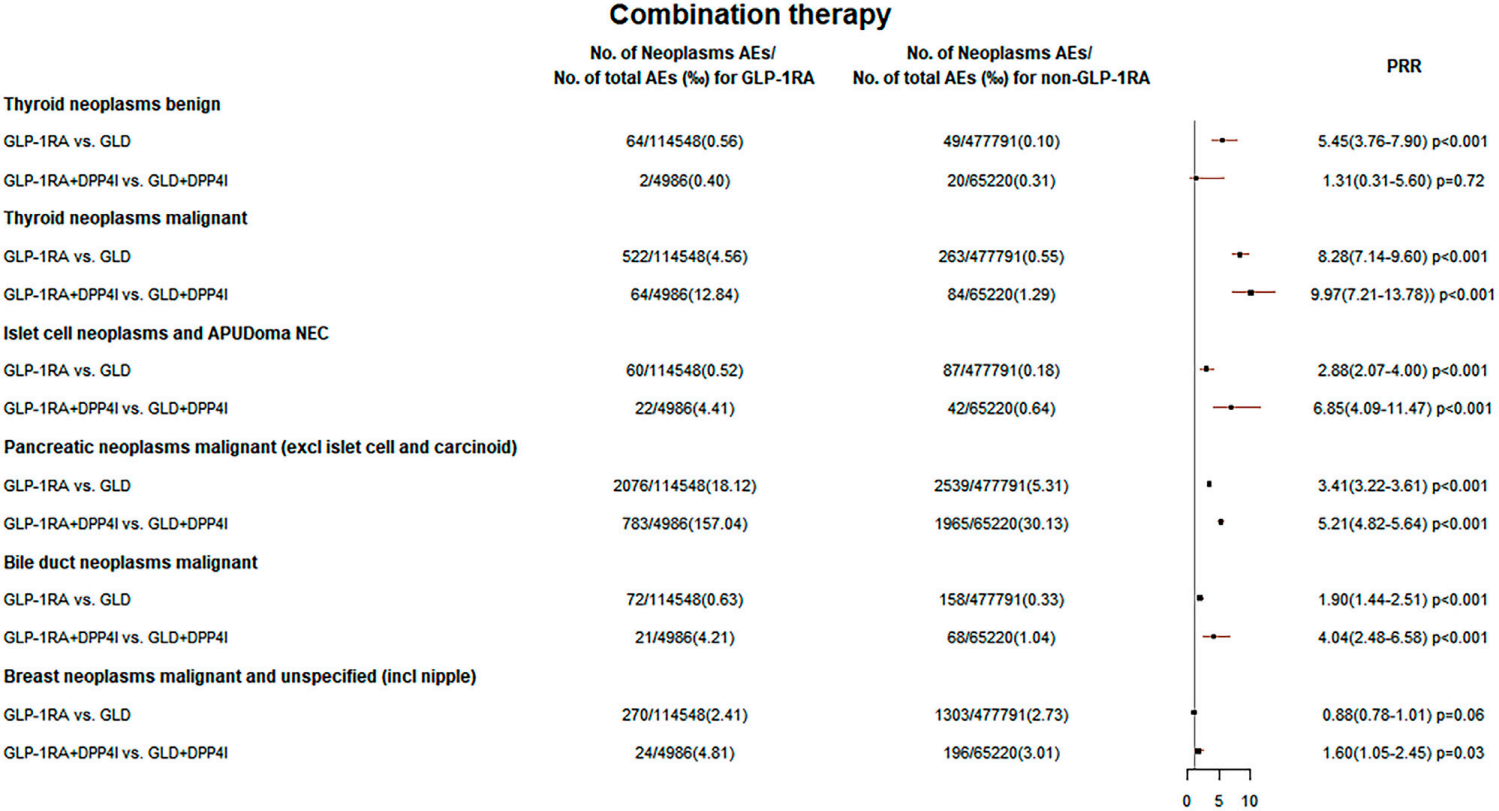
Neoplasm reporting rate of GLP-1RA and DPP4i combination therapy. The reporting rates of GLP-1RA-associated neoplasms were compared with different comparators in “GLP-1RA vs. GLD”: other glucose-lowering drugs when the indication is limited to diabetes while excluding all tumors and in “GLP-1RA + DPP4i vs. GLD + DPP4i”: only the cases in combination with DPP4i on the basis of “GLP-1RA vs. GLD” for comparison were selected.

Among pancreatic neoplasms malignant (1756 cases) and ICN&AN (56 cases) cases combined with glucose-lowering drugs (GLDs), DPP4i combined with GLP-1RA account for about 1/2 (pancreatic neoplasms malignant: 954 cases, 54.3%; ICN&AN: 25 cases, 44.6%), as shown in Supplementary Tables S3, S4. Figure 4 indicates that compared with other hypoglycemic agents in AERSMine, PRR was increased when GLP-1RA was combined with DPP4i (pancreatic neoplasms malignant: GLP-1RA vs. GLD: PRR 3.41; GLP-1RA + DPP4i vs. GLD + DPP4i: PRR 5.21; ICN&AN: GLP-1RA vs. GLD: PRR 2.88; GLP-1RA + DPP4i vs. GLD + DPP4i: PRR 6.85). GLP-1RA binding to GLP-1R, which is mainly expressed in pancreatic islets, can promote insulin synthesis and secretion, stimulate β-cell proliferation, and inhibit apoptosis (McIntosh et al., 2010). DPP4i could prevent the inactivation of GLP-1 and prolong its half-life (Overbeek et al., 2018). Thus, the combination of GLP-1RA and DPP4i may increase the risk of tumor formation. This combination therapy is not recommended in clinics, but it may occur in some free clinics with a limited choice of drugs (Lajthia et al., 2019). The findings from Figure 4 demonstrated that the PRR for thyroid neoplasms malignant was also increased in this combination therapy.

### Glucagon-like peptide-1 receptor agonist-related other neoplasms

**FIGURE 5:**
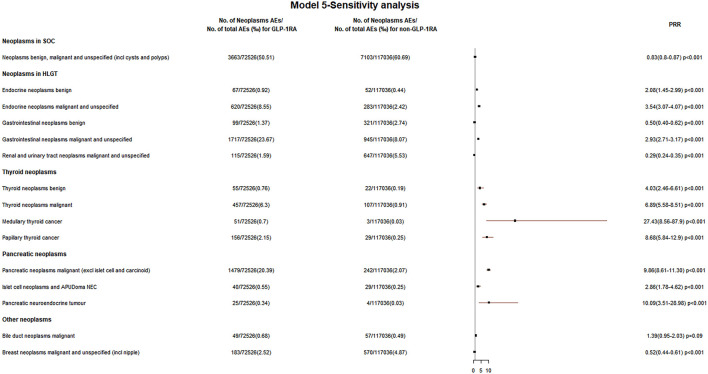
Neoplasm reporting rate in model 5—sensitivity analysis. The neoplasms associated with GLP-1RA compared with other drugs excluding GLP-1RA (non-GLP-1RA) after excluding all cases with other AEs (retinal adverse events, acute kidney injury, hypoglycemia, nausea, vomiting, diarrhea, and pancreatitis) on the basis of model 4 during 2004 Q1–2021 Q3.

Supplementary Figure S6 and Figure 5 demonstrate that the lower limit of PRR for GLP-1RA-associated bile duct cancer was greater than 1 in the first three models but less than 1 in model 4 (PRR 1.42, 95% CI 0.96–2.10, *p* = 0.03) and model 5 (PRR 1.39, 95% CI 0.95–2.03, *p* = 0.09). Moreover, Figure 4 shows that the PRR (PRR 4.04, 95% CI 2.48–6.58, *p* < 0.001) was higher when GLP-1RA was combined with DPP4i. A cohort study showed that the GLP-1RA group had a higher bile duct cancer reporting rate than sulfonylurea (ROR: 1.63, 95% CI 1.00–2.66) and thiazolidinedione (ROR: 4.73, 95% CI 2.95–7.58) groups (Abrahami et al., 2018). GLP-1R was found to be expressed in bile duct cells, and the expression was increased in cholestasis. Exenatide could stimulate bile duct cell proliferation (Marzioni et al., 2007) and reduce the apoptosis of bile duct epithelium (Marzioni et al., 2009), which are thought to be possibly related to tumor formation. However, Ueda et al. reported that neither DPP4i (HR 1.15, 95% CI 0.90–1.46) nor GLP-1RA (HR 1.25, 95% CI 0.89–1.76) increased cholangiocarcinoma risk (Ueda et al., 2021).

Supplementary Figure S6 and Figure 5 suggest that GLP-1RA did not increase the breast cancer reporting rate in five models. A meta-analysis including 52 RCTs suggested that GLP-1RA did not increase the risk of breast cancer (RR 0.98) (Piccoli et al., 2021). However, another recent study showed that the immunoreactivity of GLP-1R was significantly higher in breast cancer tissues of diabetic patients (Hashimoto Takigami et al., 2021). Figure 4 indicates that the combination of GLP-1RA with DPP4i increased the breast cancer reporting rate (PRR 1.60, 95% CI: 1.05–2.45, *p* = 0.03).

### The disproportionality analysis for different types of glucagon-like peptide-1 receptor agonists

Supplementary Figure S7 shows the PRR in model 5 for different types of GLP-1RA associated with thyroid neoplasms benign and malignant, pancreatic neoplasms malignant, and ICN&AN. Also, Supplementary Table S6 demonstrates the percentage of different brand names of GLP-1RA in the aforementioned AE patients using the FAERS public dashboard. We found that the PRR for the aforementioned four types of tumors associated with exenatide/liraglutide and the first three types of tumors associated with semaglutide were greater than 1. First, clinicians may prefer liraglutide and semaglutide for weight management in overweight patients with diabetes because liraglutide (Astrup et al., 2009; Pi-Sunyer et al., 2015) and semaglutide (Wilding et al., 2021) have been approved for the pharmacological treatment of obesity. However, obesity and insulin resistance induced by obesity are important risk factors for cancer. Then, exenatide and liraglutide were the first two GLP-1RAs to be approved for use. The warnings that GLP-1RA may be associated with an increased risk of MTC may prompt more exenatide/liraglutide-associated tumor reports to be submitted to the FDA during the long marketing period.

### Semaglutide-related neoplasms

In our study, the PRR of semaglutide-associated overall tumor was less than 1 (PRR for models 1–5: 0.39, 0.36, 0.30, 0.29, and 0.59). In the FAERS database, there were 179 tumors (2.4%) of 7461 semaglutide-related AEs, and the most common was pancreatic carcinoma (27 cases; model 5 PRR: 3.03, 95% CI 1.61–5.73, *p* < 0.01), followed by thyroid cancer (18 cases; model 5: PRR 1.57, 95% CI 0.39–6.42, *p* = 0.52), breast cancer (12 cases; model 5 PRR: 1.00, 95% CI 0.45–2.25, *p* = 1.00), pancreatic carcinoma metastatic (9 cases; model 5 PRR: 6.21, 95% CI 2.21–17.45, *p* < 0.01), and MTC (8 cases; PRR 108.62, 95% CI 27.18–434.04, *p* < 0.01). These data are shown in Supplementary Table S5. We did not find the increased PRR of semaglutide-associated overall tumor reporting rate. Among the tumor types with higher reporting rates, the disproportionality analysis of semaglutide was similar to the overall GLP-1RA. However, in some RCTs conducted on NASH (Newsome et al., 2021) and overweight and obese patients (Rubino et al., 2021; Wadden et al., 2021), the malignancy reporting rates of semaglutide were higher than those of the control groups. However, tumor patients were excluded only based on their medical history in the exclusion criteria of these RCTs; meanwhile, basal and squamous cell skin cancer and any carcinoma *in situ* can be included in STEP 3 (Wadden et al., 2021) and STEP 4 (Rubino et al., 2021). These might cause bias in the AE reports.

### Limitations

In spontaneous reports, some cases were not confirmed by healthcare professionals and reporters could decide the primary suspect drugs themselves. The causal relationship between AEs and drugs could not be proved either. Meanwhile, we cannot obtain data on patients’ physical status and insulin resistance state, the duration and dose of agents, family history, the body mass index (BMI), and personal history of alcohol abuse and smoking, which may influence the development of tumors. The follow-up information is generally not included in these reports, and mortality rates may be underestimated.

In this study, not all tumors were disproportionately analyzed at the PT level. We conducted disproportionality analysis at the SOC/HGLT/HLT levels and discussed the HLT-level tumors in which PRR was robustly greater than 1. For the PT level, some tumors were analyzed including MTC and PTC as the association of GLP-1RA with these two tumors has attracted widespread attention; meanwhile, the disproportionality analysis of pancreatic neuroendocrine tumors was also performed at the PT level because it had the highest proportion in islet cell tumors and APUDoma NEC. Using high-level terms to detect signals has greater sensitivity than PT/LLT level terms (Pearson et al., 2009), but higher levels are less specific and do not clearly define a precise medical condition. Since higher level terms may include some nonspecific terms, signal detection may be influenced by these nonspecific terms.

## Conclusion

Concerns about safety issues, especially in oncology AEs have arisen due to the widespread use of GLP-1RA. Despite existing limitations, spontaneous reporting may be the best way to find rare AEs. Our findings provided new real-world evidence for oncology safety information of GLP-1RA. By mining the FAERS database, we comprehensively and systematically analyzed the relationship between GLP-1RA and tumors and did not find abnormally elevated GLP-1RA associated with overall tumor cases at the SOC level and most types of tumor cases at the HLGT/HLT levels. Disproportionality analyses found that GLP-1RA was associated with thyroid neoplasms benign and malignant, pancreatic neoplasms malignant, and ICN&AN cases. In thyroid cancers, in addition to MTC, the reporting rate of PTC also increased. Also, the combination of GLP-1RA with DPP4i perhaps increases some tumor reporting rates, including thyroid neoplasms malignant, pancreatic neoplasms malignant, ICN&AN, bile duct cancer, and breast cancer. However, given the cardiac and renal benefits as well as the weight loss effect of GLP-1RA, the increased PRR does not mean that clinicians should restrict the use of GLP-1RA but should remain appropriate vigilance for potential adverse effects.

## Data Availability

Publicly available datasets were analyzed in this study. These data can be found at: 1) https://research.cchmc.org/aers/explore.jsp 2) https://fis.fda.gov/sense/app/95239e26-e0be-42d9-a960-9a5f7f1c25ee/sheet/7a47a261-d58b-4203-a8aa-6d3021737452/state/analysis.

## Abbreviations

ICN&AN, islet cell neoplasms and APUDoma NEC; MTC, medullary thyroid cancer; PTC, papillary thyroid cancer; GLD, glucose-lowering drug.
