# Supplementary material for: GLP-1 receptor agonist-associated tumor adverse events: A real-world study from 2004 to 2021 based on FAERS
Source: Front Pharmacol. 2022 Oct 25;13:925377. doi: 10.3389/fphar.2022.925377 (PMC9640975; doi:10.3389/fphar.2022.925377)
Supplement: Supplementary file 1 [file DataSheet2.docx]

Supplementary Material 2

Table S1. The case details of thyroid neoplasms benign

| **Variable** | **Available number** | **Value** |
| --- | --- | --- |
| Total reports, n (%) | 85 (100%) |  |
| **Demographic characteristics** |  |  |
| Age, n (%) | 58 (68.2%) |  |
| Mean ± SD (range), (years) |  | 58.9±10.0 (40-83) |
| Sex, n (%) | 82 (96.5%) |  |
| Male |  | 20 (24.4%) |
| Weight, n (%) | 38 (44.7%) |  |
| Mean ± SD (range), (kg) |  | 100.6±22.1 (70-181) |
| **Concomitant hypoglycemic drugs, n (%)** | 29 (34.1%) |  |
| Sulphonylurea |  | 10 (34.5%) |
| Metformin |  | 17 (58.6%) |
| α-glycosidase inhibitor |  | 0 (-) |
| Thiazolidinediones |  | 0 (-) |
| DPP4i |  | 2 (6.9%) |
| SGLT2i |  | 1 (3.4%) |
| Insulin |  | 7(24.1%) |
| **Concomitant other drugs, n (%)*** | 41 (48.2%) |  |
| Synthroid |  | 8 (19.5%) |
| Aspirin |  | 6 (14.6%) |
| Crestor |  | 5 (12.2%) |
| Lipitor |  | 5 (12.2%) |
| Gabapentin |  | 4 (9.8%) |
| Metoprolol |  | 4 (9.8%) |
| Simvastatin |  | 4 (9.8%) |
| Pantoprazole |  | 4 (9.8%) |
| **Concomitant complications, n (%)*** | 80 (94.1%) |  |
| Type 2 Diabetes Mellitus |  | 45 (56.3%) |
| Product Used For Unknown Indication |  | 19 (23.8%) |
| Diabetes Mellitus |  | 13 (16.3%) |
| Obesity |  | 7 (8.8%) |
| Hypertension |  | 5 (6.3%) |
| **Reaction, n (%)** | 85 (100%) |  |
| Benign neoplasm of thyroid gland |  | 47 (55.3%) |
| Haemorrhagic thyroid cyst |  | 0 (-) |
| Thyroglossal cyst |  | 2 (2.4%) |
| Thyroid adenoma |  | 15 (17.7%) |
| Thyroid cyst |  | 21 (24.7%) |
| **Outcome, n (%)** | 85 (100%) |  |
| Hospitalization |  | 22 (25.9%) |
| Disability |  | 0 (-) |
| Life threatening |  | 1 (1.2%) |
| Death |  | 0 (-) |
| **Reporting source, n (%)** | 85 (100%) |  |
| Health care professionals |  | 46 (54.1%) |
| Non-health care professionals |  | 39 (45.9%) |
| **Reporting country, n (%)** | 80 (94.1%) |  |
| U.S. |  | 63 (78.8%) |
| Outside U.S. |  | 17 (21.3%) |

Table S2. The case details of thyroid neoplasms malignant

| **Variable** | **Available number** | **Value** |
| --- | --- | --- |
| Total reports, n (%) | 698 (100%) |  |
| **Demographic characteristics** |  |  |
| Age, n (%) | 389 (55.7%) |  |
| Mean ± SD (range), (years) |  | 50.9±16.2 (20-83) |
| Sex, n (%) | 630 (90.3%) |  |
| Male |  | 195 (31.0%) |
| Weight, n (%) | 278 (39.8%) |  |
| Mean ± SD (range), (kg) |  | 102.7±24.6 (46-191.8) |
| **Concomitant hypoglycemic drugs, n (%)** | 273 (39.1%) |  |
| Sulphonylurea |  | 77 (28.2%) |
| Metformin |  | 194 (71.1%) |
| α-glycosidase inhibitor |  | 2 (0.7%) |
| Thiazolidinediones |  | 7 (2.6%) |
| DPP4i |  | 46 (16.8%) |
| SGLT2i |  | 4 (1.5%) |
| Insulin |  | 82 (30.0%) |
| **Concomitant other drugs, n (%)*** | 312 (44.7%) |  |
| Synthroid |  | 84 (26.9%) |
| Aspirin |  | 66 (21.2%) |
| Lisinopril |  | 53 (17.0%) |
| Levothyroxine |  | 42 (13.5%) |
| Hydrochlorothiazide |  | 36 (11.5%) |
| Simvastatin |  | 36 (11.5%) |
| **Concomitant complications, n (%)*** | 572 (81.9%) |  |
| Type 2 Diabetes Mellitus |  | 385 (67.3%) |
| Product Used For Unknown Indication |  | 175 (30.6%) |
| Diabetes Mellitus |  | 51 (8.9%) |
| Hypertension |  | 29 (5.1%) |
| Hypothyroidism |  | 15 (2.6%) |
| **Reaction, n (%)** | 698 (100%) |  |
| Anaplastic thyroid cancer |  | 4 (0.6%) |
| Follicular thyroid cancer |  | 16 (2.3%) |
| Huerthle cell carcinoma |  | 1 (0.1%) |
| Medullary thyroid cancer |  | 77 (11.0%) |
| Papillary thyroid cancer |  | 202 (28.9%) |
| Poorly differentiated thyroid carcinoma |  | 0 (-) |
| Thyroid cancer |  | 698 (100%) |
| Thyroid cancer recurrent |  | 14 (2.0%) |
| **Outcome, n (%)** | 698 (100%) |  |
| Hospitalization |  | 95 (13.6%) |
| Disability |  | 14 (2.0%) |
| Life threatening |  | 17 (2.4%) |
| Death |  | 27 (3.9%) |
| **Reporting source, n (%)** | 695 (99.6%) |  |
| Health care professionals |  | 340 (48.9%) |
| Non-health care professionals |  | 355 (51.1%) |
| **Reporting country, n (%)** | 675 (96.7%) |  |
| U.S. |  | 604 (89.5%) |
| Outside U.S. |  | 71 (10.5%) |

Table S3. The case details of pancreatic neoplasms malignant (excl islet cell and carcinoid)

| **Variable** | **Available number (%)** | **Value** |
| --- | --- | --- |
| Total reports, n (%) | 2910 (100%) |  |
| **Demographic characteristics** |  |  |
| Age, n (%) | 1541 (53.0%) |  |
| Mean ± SD (range), (years) |  | 64.1±9.0 (34-91) |
| Sex, n (%) | 2724 (94.6%) |  |
| Male | 1591 (58.4%) |  |
| Weight, n (%) | 1176 (43.2%) |  |
| Mean ± SD (range), (kg) |  | 93.7±24.6 (20-261.1) |
| **Concomitant hypoglycemic drugs, n (%)** | 1756 (60.3%) |  |
| Sulphonylurea |  | 305 (17.4%) |
| Metformin |  | 944 (53.8%) |
| α-glycosidase inhibitor |  | 4 (0.2%) |
| Thiazolidinediones |  | 23 (1.3%) |
| DPP4i |  | 954 (54.3%) |
| SGLT2i |  | 30 (1.7%) |
| Insulin |  | 500 (28.5%) |
| **Concomitant other drugs, n (%)*** | 1234 (42.4%) |  |
| Aspirin |  | 308 (25.0%) |
| Lisinopril |  | 192 (15.6%) |
| Lipitor |  | 145 (11.8%) |
| Simvastatin |  | 142 (11.5%) |
| Actos |  | 132 (10.7%) |
| **Concomitant complications, n (%)*** | 2288 (78.6%) |  |
| Type 2 Diabetes Mellitus |  | 1553 (67.9%) |
| Product Used For Unknown Indication |  | 649 (28.4%) |
| Diabetes Mellitus |  | 264 (11.5%) |
| Hypertension |  | 160 (7.0%) |
| Pain |  | 81 (3.5%) |
| **Reaction, n (%)** | 2910 (100%) |  |
| Acinar cell carcinoma of pancreas |  | 5 (0.2%) |
| Adenocarcinoma pancreas |  | 264 (9.1%) |
| Cystadenocarcinoma pancreas |  | 4 (0.1%) |
| Ductal adenocarcinoma of pancreas |  | 18 (0.6%) |
| Intraductal papillary-mucinous carcinoma of pancreas |  | 11 (0.4%) |
| Pancreatic carcinoma |  | 2650 (91.1%) |
| Pancreatic carcinoma metastatic |  | 589 (20.1%) |
| Pancreatic carcinoma recurrent |  | 13 (0.5%) |
| Pancreatic sarcoma |  | 0 (-) |
| Pancreatoblastoma |  | 0 (-) |
| Solid pseudopapillary tumour of the pancreas |  | 1 (0.03%) |
| **Outcome, n (%)** | 2910 (100%) |  |
| Hospitalization |  | 876 (30.1%) |
| Disability |  | 112 (3.9%) |
| Life threatening |  | 272 (9.4%) |
| Death |  | 1595 (54.8%) |
| **Reporting source, n (%)** | 2904 (99.8%) |  |
| Health care professionals |  | 1386 (47.6%) |
| Non-health care professionals |  | 1518 (52.2%) |
| **Reporting country, n (%)** | 2845 (97.8%) |  |
| U.S. |  | 2316 (81.4%) |
| Outside U.S. |  | 529 (18.6%) |

Table S4. The case details of islet cell neoplasms and APUDoma NEC

| **Variable** | **Available number (%)** | **Value** |
| --- | --- | --- |
| Total reports, n (%) | 78 (100%) |  |
| **Demographic characteristics** |  |  |
| Age, n (%) | 57 (73.1%) |  |
| Mean ± SD (range), (years) |  | 59.1±9.3 (32-84) |
| Sex, n (%) | 76 (97.4%) |  |
| Male | 42 (55.3%) |  |
| Weight, n (%) | 53 (69.7%) |  |
| Mean ± SD (range), (kg) |  | 103.5±21.3 (62.6-159.2) |
| **Concomitant hypoglycemic drugs, n (%)** | 56 (71.8%) |  |
| Sulphonylurea |  | 16 (28.6%) |
| Metformin |  | 41 (73.2%) |
| α-glycosidase inhibitor |  | 0 (-) |
| Thiazolidinediones |  | 3 (5.4%) |
| DPP4i |  | 25 (44.6%) |
| SGLT2i |  | 3 (5.4%) |
| Insulin |  | 11 (19.6%) |
| **Concomitant other drugs, n (%)*** | 54 (69.2%) |  |
| Simvastatin |  | 12 (22.2%) |
| Actos |  | 10 (18.5%) |
| Amaryl |  | 7 (13.0%) |
| Lipitor |  | 7 (13.0%) |
| Aspirin |  | 7 (13.0%) |
| Hydrochlorothiazide |  | 7 (13.0%) |
| Oxycodone |  | 7 (13.0%) |
| **Concomitant complications, n (%)*** | 65 (83.3%) |  |
| Type 2 Diabetes Mellitus |  | 36 (55.4%) |
| Product Used For Unknown Indication |  | 10 (15.4%) |
| Diabetes Mellitus |  | 5 (7.7%) |
| Hypertension |  | 4 (6.2%) |
| Goitre |  | 3 (4.6%) |
| **Reaction, n (%)** | 78 (100%) |  |
| APUDoma |  | 0 (-) |
| Gastrinoma |  | 3 (3.9%) |
| Gastrinoma malignant |  | 0 (-) |
| Gastroenteropancreatic neuroendocrine tumour disease |  | 1 (1.3%) |
| Glucagonoma |  | 0 (-) |
| Insulinoma |  | 4 (5.1%) |
| Malignant neoplasm of islets of Langerhans |  | 2 (2.6%) |
| Metastatic glucagonoma |  | 0 (-) |
| Neurotensinoma |  | 0 (-) |
| Pancreatic neuroendocrine tumour |  | 67 ( 85.9%) |
| Pancreatic neuroendocrine tumour metastatic |  | 19 (24.4%) |
| Phaeochromocytoma |  | 2 (2.6%) |
| Phaeochromocytoma crisis |  | 0 (-) |
| Phaeochromocytoma malignant |  | 0 (-) |
| Somatostatinoma |  | 0 (-) |
| Vipoma |  | 0 (-) |
| **Outcome, n (%)** | 78(100%) |  |
| Hospitalization |  | 38 (48.7%) |
| Disability |  | 5 (6.4%) |
| Life threatening |  | 10 (12.8%) |
| Death |  | 14 (18.0%) |
| **Reporting source, n (%)** | 78 (100%) |  |
| Health care professionals |  | 58 (74.4%) |
| Non-health care professionals |  | 20 (25.6%) |
| **Reporting country, n (%)** | 77 (98.7%) |  |
| U.S. |  | 56 (75.3%) |
| Outside U.S. |  | 21 (27.3%) |

Table1-4: As not all reports are complete, n (%) refers to the number and percentage of reports available for each variable.

DPP4i: Dipeptidyl peptidase 4 (DPP-4) inhibitors; SGLT2i: sodium glucose cotransporter-2 inhibitors

Table S5. Semaglutide-associated neoplasms cases

| **Neoplasms in soc** | **Neoplasms in PT** | **Number** |
| --- | --- | --- |
| Respiratory and mediastinal neoplasms benign (excl mesotheliomas) | Laryngeal Cyst | 1 |
| Respiratory and mediastinal neoplasms malignant and unspecified | Laryngeal cancer | 1 |
|  | Lung cancer metastatic | 1 |
| Breast neoplasms malignant and unspecified (incl nipple) | Breast Cancer | 12 |
|  | Breast Cancer Metastatic | 1 |
| Endocrine neoplasms benign | Adrenal Adenoma | 2 |
|  | Pituitary Tumour Benign | 1 |
|  | Benign neoplasm of thyroid gland | 4 |
|  | Thyroid cyst | 1 |
| Endocrine neoplasms malignant and unspecified | Neuroendocrine carcinoma | 1 |
|  | Neuroendocrine tumour | 6 |
|  | Pituitary tumour | 2 |
|  | Thyroid neoplasm | 5 |
|  | Pancreatic neuroendocrine tumour | 3 |
|  | Medullary thyroid cancer | 8 |
|  | Thyroid cancer | 18 |
| Gastrointestinal neoplasms benign | Intraductal papillary mucinous neoplasm | 2 |
|  | Pancreatic cyst | 8 |
|  | Colon adenoma | 1 |
|  | Gastric polyps | 3 |
| Gastrointestinal neoplasms malignant and unspecified | Colorectal cancer metastatic | 1 |
|  | Gastric cancer | 2 |
|  | Gastric neoplasm | 1 |
|  | Pancreatic neoplasm | 3 |
|  | Gastrointestinal cancer metastatic | 1 |
|  | Gastrointestinal carcinoma | 1 |
|  | Adenocarcinoma pancreas | 2 |
|  | Intraductal papillary-mucinous carcinoma of pancreas | 2 |
|  | Pancreatic carcinoma | 27 |
|  | Pancreatic carcinoma metastatic | 9 |
|  | Small intestine carcinoma | 1 |
| Hepatic and biliary neoplasms benign | Gallbladder polyp | 1 |
| Hepatobiliary neoplasms malignant and unspecified | Cholangiocarcinoma | 2 |
|  | Hepatic cancer | 2 |
|  | Hepatocellular carcinoma | 1 |
| Renal and urinary tract neoplasms benign | Renal adenoma | 2 |
|  | Renal cyst | 2 |
| Renal and urinary tract neoplasms malignant and unspecified | Bladder Cancer Recurrent | 1 |
|  | Bladder Cancer | 2 |
| Reproductive neoplasms female benign | Haemorrhagic Ovarian Cyst | 1 |
| Reproductive neoplasms female malignant and unspecified | Ovarian Cancer | 1 |
|  | Endometrial adenocarcinoma | 1 |
|  | Endometrial Cancer | 1 |
|  | Uterine Cancer | 1 |
| Reproductive neoplasms male malignant and unspecified | Prostate cancer | 1 |
| Cutaneous neoplasms benign | Melanocytic naevus | 1 |
| Skin neoplasms malignant and unspecified | Malignant melanoma | 1 |
|  | Basal Cell Carcinoma | 1 |
|  | Neuroendocrine carcinoma of the skin | 1 |
|  | Skin cancer | 2 |
| Soft tissue neoplasms benign | Lipoma | 1 |
| Soft tissue neoplasms malignant and unspecified | Neurofibrosarcoma | 1 |
| Haematopoietic neoplasms (excl leukaemias and lymphomas) | Splenic cyst | 1 |
| Leukaemias | Acute myeloid leukaemia | 1 |
|  | Chronic lymphocytic leukaemia | 1 |
|  | Myeloid leukaemia | 1 |
| Lymphomas NEC | Lymphoma | 3 |
| Lymphomas non-Hodgkin's B-cell | Diffuse Large B-Cell Lymphoma Stage Iii | 1 |
| Lymphomas non-Hodgkin's T-cell | T-cell lymphoma | 1 |
|  | Cutaneous T-Cell Lymphoma | 1 |
|  | Peripheral T-Cell Lymphoma Unspecified | 1 |
| Lymphomas non-Hodgkin's unspecified histology | Non-Hodgkin'S Lymphoma | 1 |
| Plasma cell neoplasms | Plasma Cell Myeloma | 1 |
| Metastases | Metastases to adrenals | 1 |
|  | Metastases to bone | 1 |
|  | Metastases To Liver | 3 |
|  | Metastases To Lymph Nodes | 1 |
|  | Metastases To Lung | 1 |
| Miscellaneous and site unspecified neoplasms benign | Cardiac myxoma | 1 |
|  | Adenoma benign | 1 |
|  | Benign Neoplasm(Thyroid Mass) | 1 |
|  | Cyst | 5 |
|  | Infected Cyst | 1 |
|  | Gastric Polyps | 3 |
|  | Polyp | 2 |
|  | Gallbladder Polyp | 1 |
| Miscellaneous and site unspecified neoplasms malignant and unspecified | Adenocarcinoma | 1 |
|  | Endometrial Adenocarcinoma | 1 |
|  | Neoplasm malignant | 7 |
|  | Basal Cell Carcinoma | 1 |
|  | Neoplasm Progression | 1 |
|  | Neoplasm | 1 |

**Table S6: The proportion for different types of GLP-1RA in with thyroid neoplasms benign and malignant, pancreatic neoplasms malignant (excl islet cell and carcinoid) and** **ICN&AN cases**

| Neoplasms | The type of GLP-1RA | Cases:N ( %) |
| --- | --- | --- |
| Thyroid neoplasms benign (Total: 85 cases) |  |  |
|  | Dulaglutide (Trulicity) | 13 (15.3%) |
|  | Exenatide extended release (Bydureon) | 11 (12.9%) |
|  | Exenatide (Byetta) | 21 (24.7%) |
|  | Semaglutide (Ozempic) | 5 (5.9%) |
|  | Semaglutide (Rybelsus) | 0 (-) |
|  | Liraglutide (Victoza) | 32 (37.6%) |
|  | Lixisenatide (Adlyxin) | 0 (-) |
| Thyroid neoplasms malignant  (Total: 698 cases) |  |  |
|  | Dulaglutide (Trulicity) | 64 (9.2%) |
|  | Exenatide extended release (Bydureon) | 35 (5.0%) |
|  | Exenatide (Byetta) | 373 (53.4%) |
|  | Semaglutide (Ozempic) | 17 (2.4%) |
|  | Semaglutide (Rybelsus) | 1 (0.1%) |
|  | Liraglutide (Victoza) | 212 (39.0%) |
|  | Lixisenatide (Adlyxin) | 0 (-) |
| pancreatic neoplasms malignant (excl islet cell and carcinoid)  (Total: 2910 cases) |  |  |
|  | Dulaglutide (Trulicity) | 202 (6.9%) |
|  | Exenatide extended release (Bydureon) | 159 (5.5%) |
|  | Exenatide (Byetta) | 1665 (57.2%) |
|  | Semaglutide (Ozempic) | 39 (1.3%) |
|  | Semaglutide (Rybelsus) | 0 (-) |
|  | Liraglutide (Victoza) | 1268 (43.6%) |
|  | Lixisenatide (Adlyxin) | 0 (-) |
| Islet cell neoplasms and APUDoma NEC  (Total: 78 cases) |  |  |
|  | Dulaglutide (Trulicity) | 7 (9.0%) |
|  | Exenatide extended release (Bydureon) | 8 (10.3%) |
|  | Exenatide (Byetta) | 46 (59.0%) |
|  | Semaglutide (Ozempic) | 3 (3.8%) |
|  | Semaglutide (Rybelsus) | 0 (-) |
|  | Liraglutide (Victoza) | 26 (33.3%) |
|  | Lixisenatide (Adlyxin) | 0 (-) |
