# Supplementary material for: GLP-1 receptor agonist-associated tumor adverse events: A real-world study from 2004 to 2021 based on FAERS
Source: Front Pharmacol. 2022 Oct 25;13:925377. doi: 10.3389/fphar.2022.925377 (PMC9640975; doi:10.3389/fphar.2022.925377)
Supplement: Supplementary file 2 [file DataSheet1.docx]

Supplementary Material 1

Figure S1. All neoplasms reporting rates in model 1.


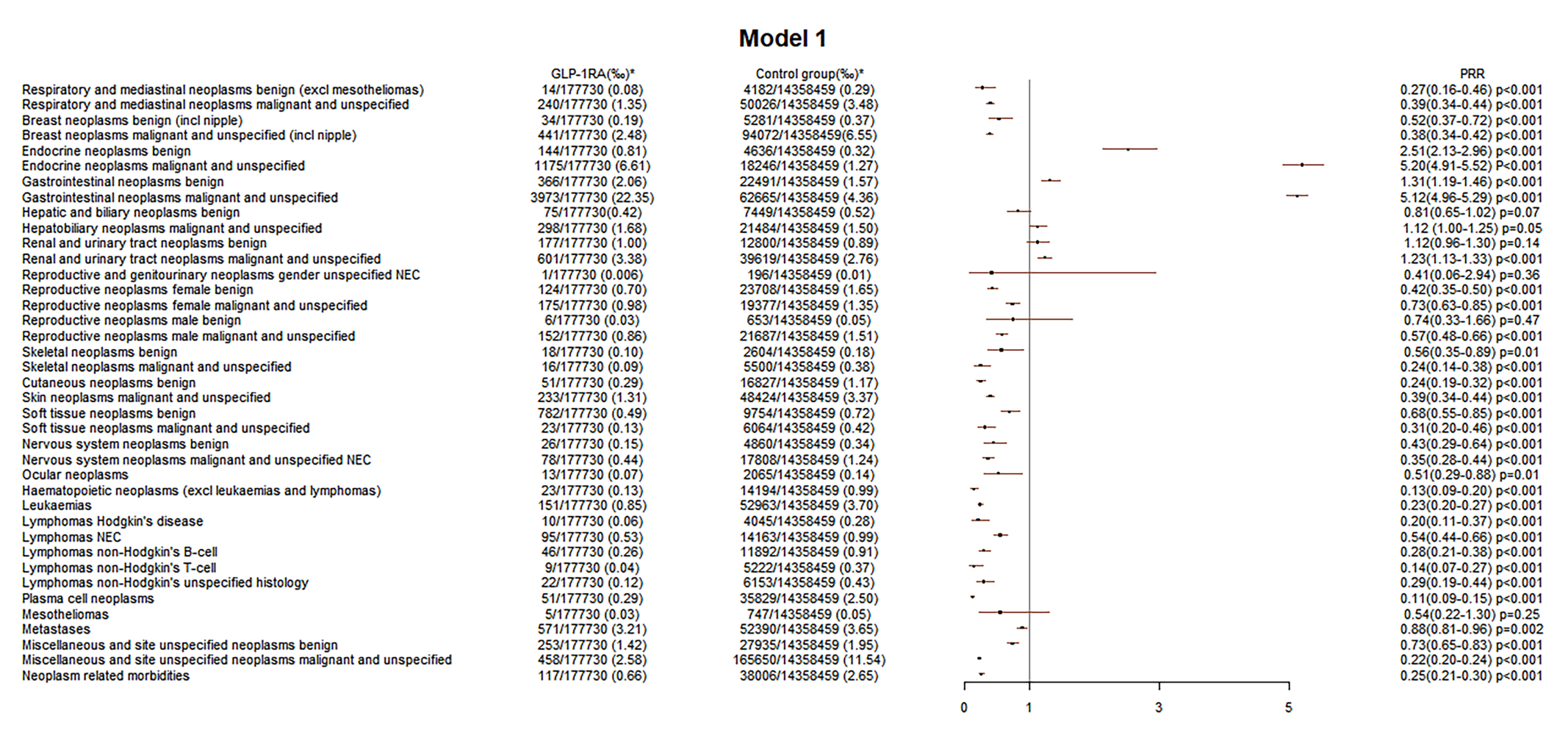


Model 1: All neoplasms in HLGT associated with GLP-1RA compared with other drugs excluding GLP-1RA (non-GLP-1RA) without indication restrictions during 2004Q1-2020Q2.

GLP-1RA (‰)*:No. of Neoplasms AEs/ No. of total AEs (‰) for GLP-1RA, Control group (‰)*:No. of Neoplasms AEs/ No. of total AEs (‰) for non-GLP-1RA

Figure S2. All neoplasms reporting rates in model 2.


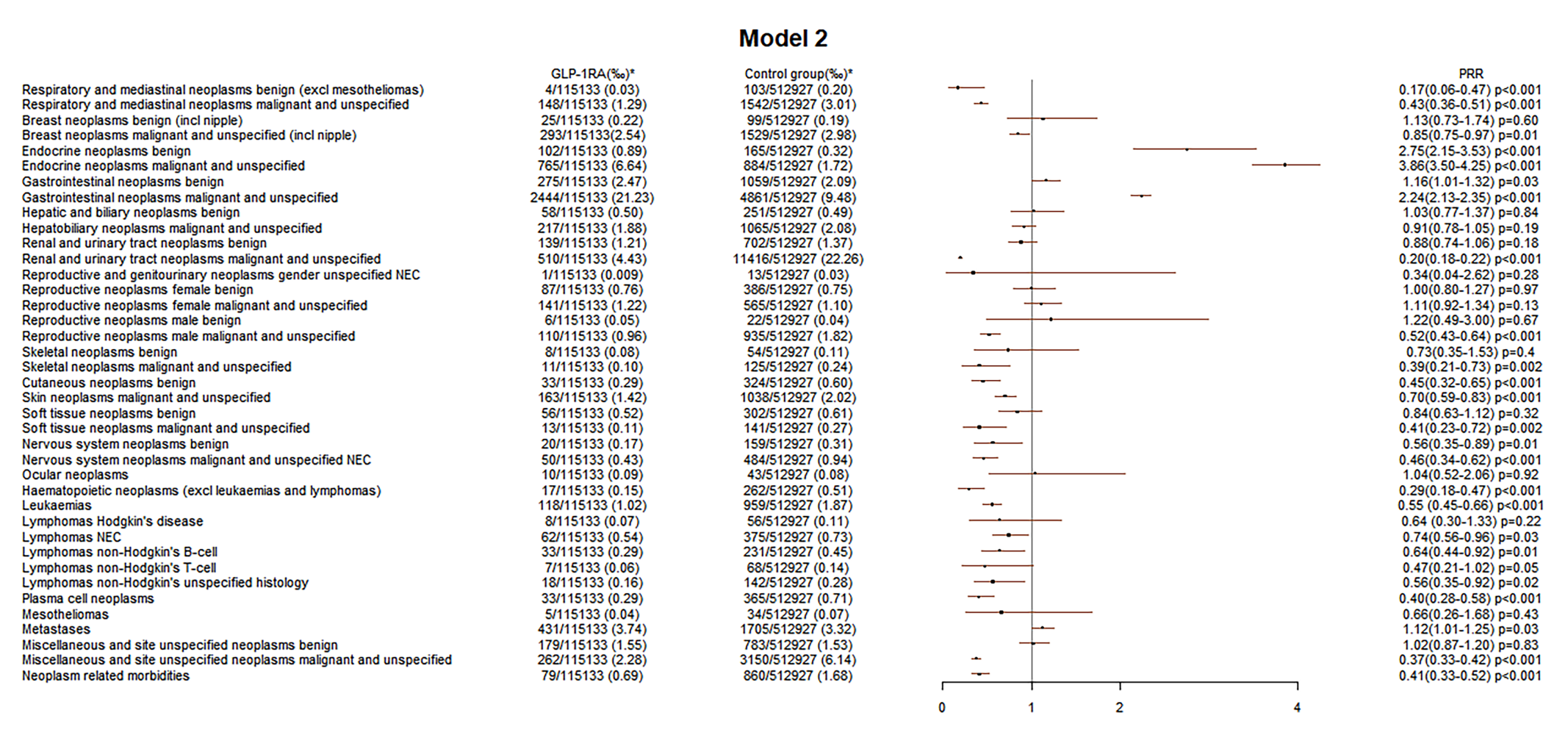
Model 2: All neoplasms in HLGT associated with GLP-1RA compared with other drugs excluding GLP-1RA (non-GLP-1RA) when diabetes as indication during 2004Q1-2020Q2.

GLP-1RA (‰)*:No. of Neoplasms AEs/ No. of total AEs (‰) for GLP-1RA, Control group (‰)*:No. of Neoplasms AEs/ No. of total AEs (‰) for non-GLP-1RA

**Figure S3. All neoplasms reporting rates in model 3.**


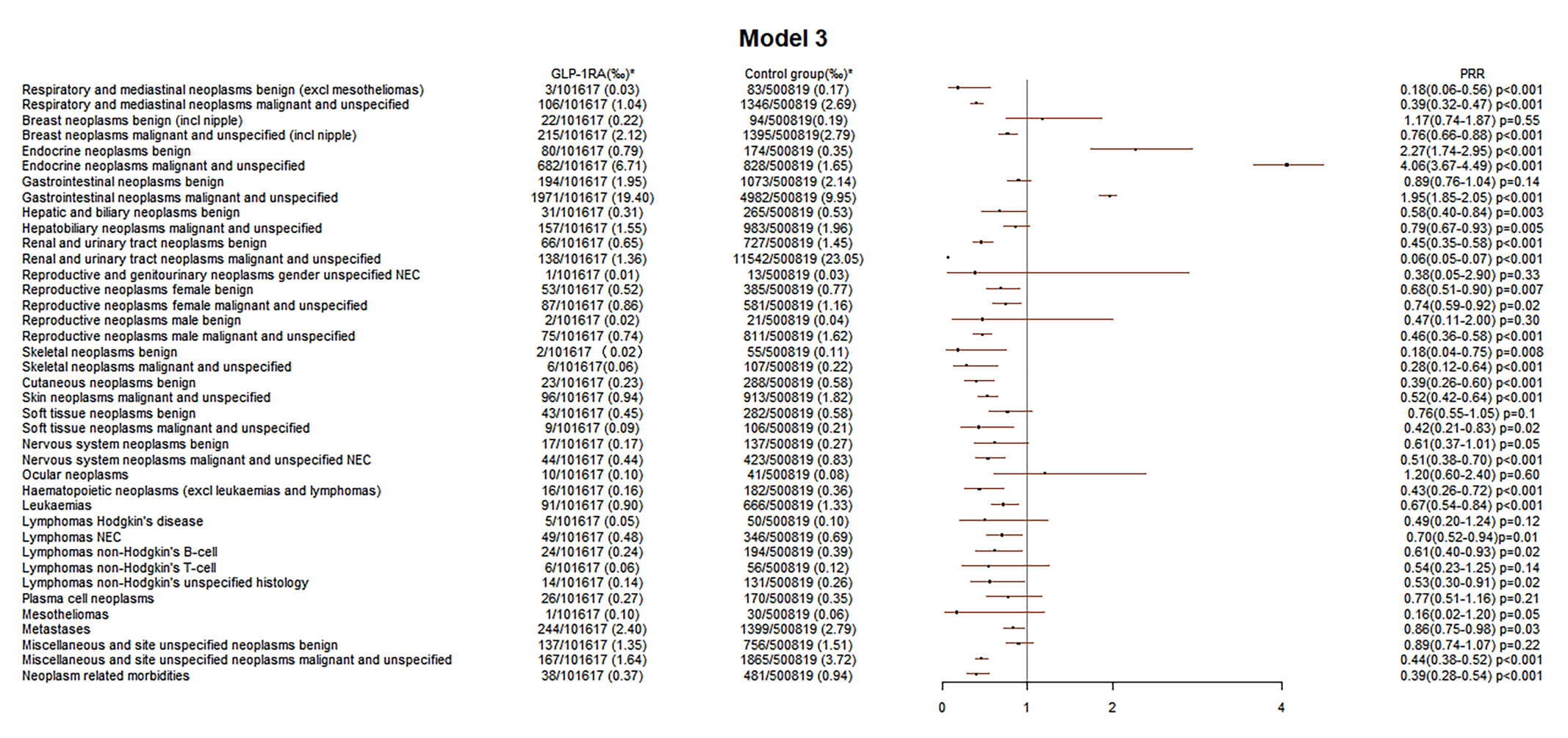


Model 3: All neoplasms in HLGT associated with GLP-1RA compared with other drugs excluding GLP-1RA (non-GLP-1RA) when the indication is limited to diabetes while excluding all tumors, and all cases with GLP-1RA as the "primary suspect" drugs during 2004Q1-2020Q2.

GLP-1RA (‰)*:No. of Neoplasms AEs/ No. of total AEs (‰) for GLP-1RA, Control group (‰)*:No. of Neoplasms AEs/ No. of total AEs (‰) for non-GLP-1RA

**Figure S4. All neoplasms reporting rates in model 4.**


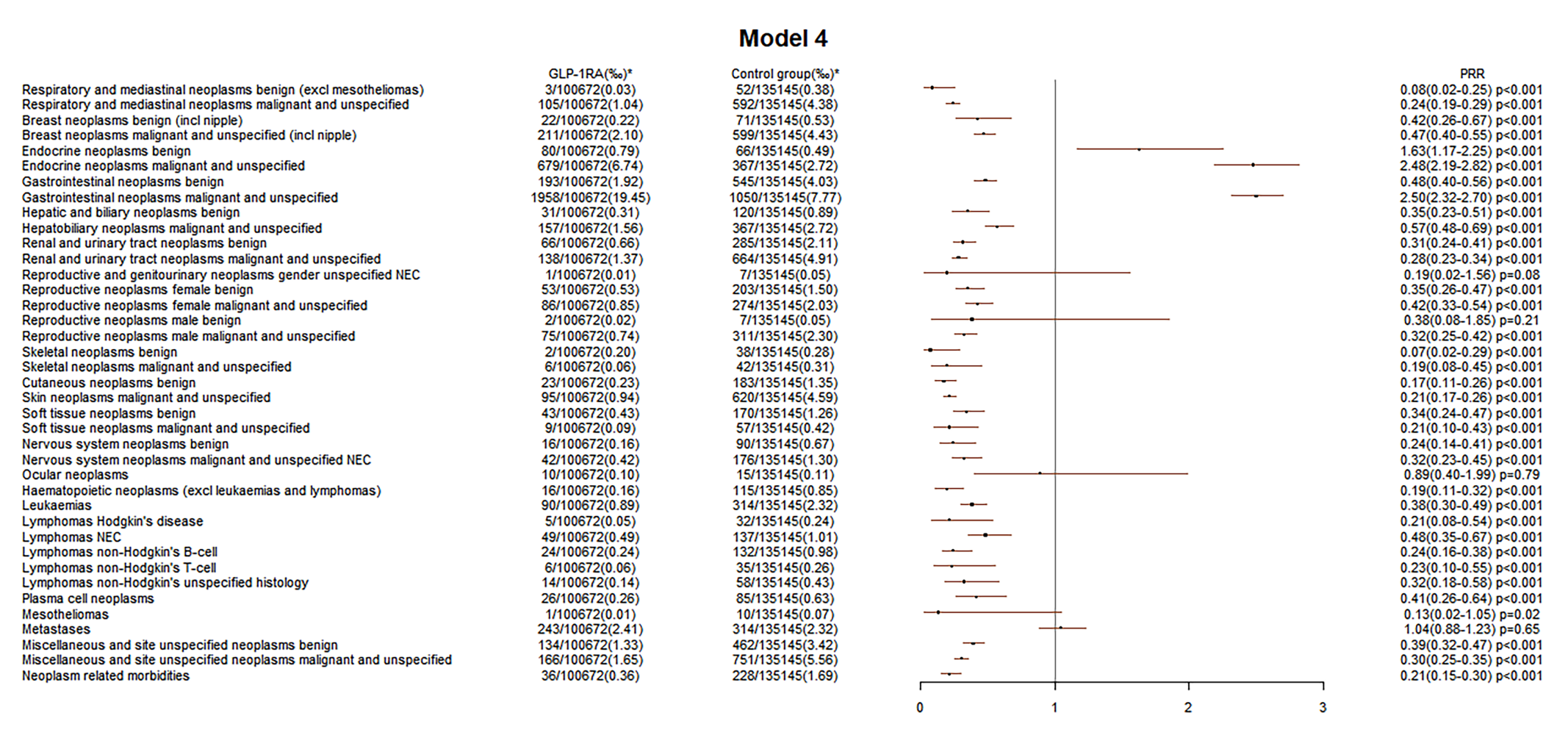
Model 4: All neoplasms in HLGT associated with GLP-1RA compared with other drugs excluding GLP-1RA (non-GLP-1RA) when all cases combining other glucose-lowering drugs were excluded on the basis of model 3 during 2004Q1-2020Q2.

GLP-1RA (‰)*:No. of Neoplasms AEs/ No. of total AEs (‰) for GLP-1RA, Control group (‰)*:No. of Neoplasms AEs/ No. of total AEs (‰) for non-GLP-1RA

**Figure S5. All neoplasms in model 5-**[**sensitivity analysis**](javascript:;)

**
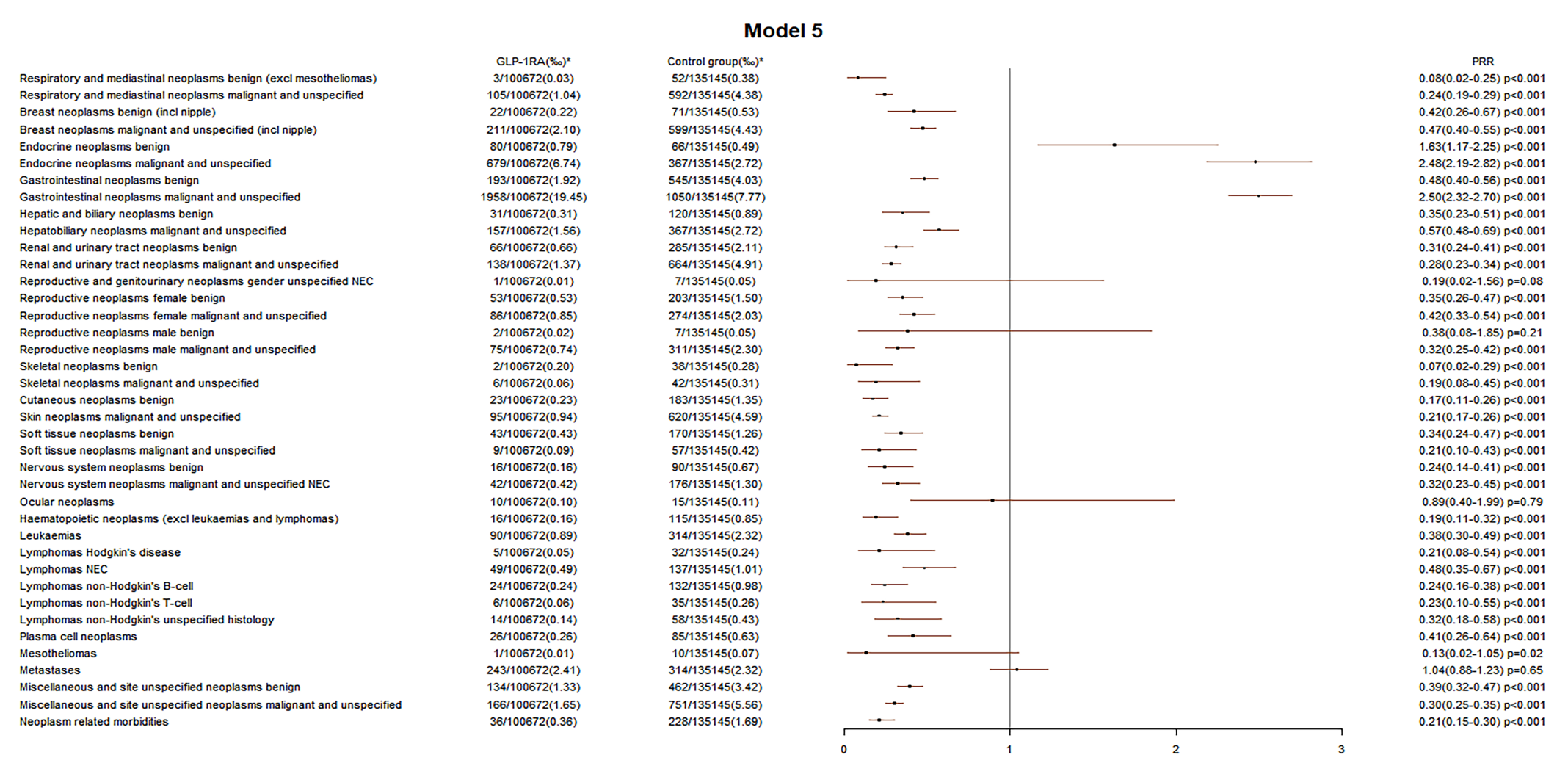
**

Model 5: All neoplasms in HLGT associated with GLP-1RA compared with other drugs excluding GLP-1RA (non-GLP-1RA) when all cases combining Other AEs (retinal adverse events, acute kidney injury, hypoglycemia, nausea, vomiting, diarrhea, and pancreatitis) were excluded on the basis of model 4 during 2004Q1-2021Q3.

GLP-1RA (‰)*:No. of Neoplasms AEs/ No. of total AEs (‰) for GLP-1RA, Control group (‰)*:No. of Neoplasms AEs/ No. of total AEs (‰) for non-GLP-1RA

Figure S6. Other neoplasms reporting rates


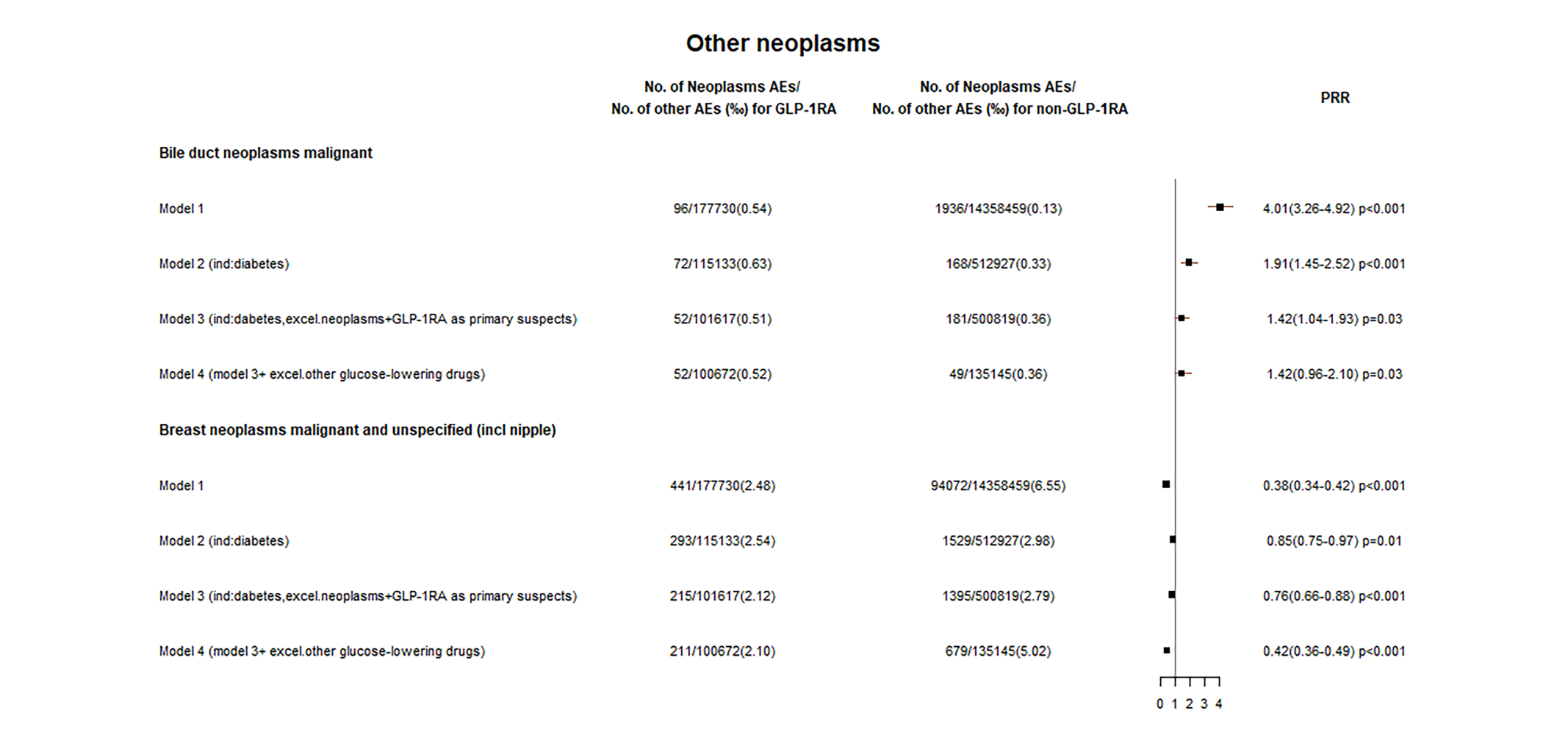


The reporting rates of GLP-1RA-associated neoplasms were compared with different comparators in 4 models during 2004Q1-2020Q2: 1) other drugs excluding GLP-1RA (non-GLP-1RA) without indication restrictions; 2) non-GLP-1RA when diabetes as indication; 3) non-GLP-1RA when the indication is limited to diabetes while excluding all tumors, and all cases with GLP-1RA as the "primary suspect" drugs. And 4) all cases combining other glucose-lowering drugs were excluded on the basis of model 3 for comparison .

**Figure S7:** **The disproportionality analysis for different types of GLP-1RA in model 5**


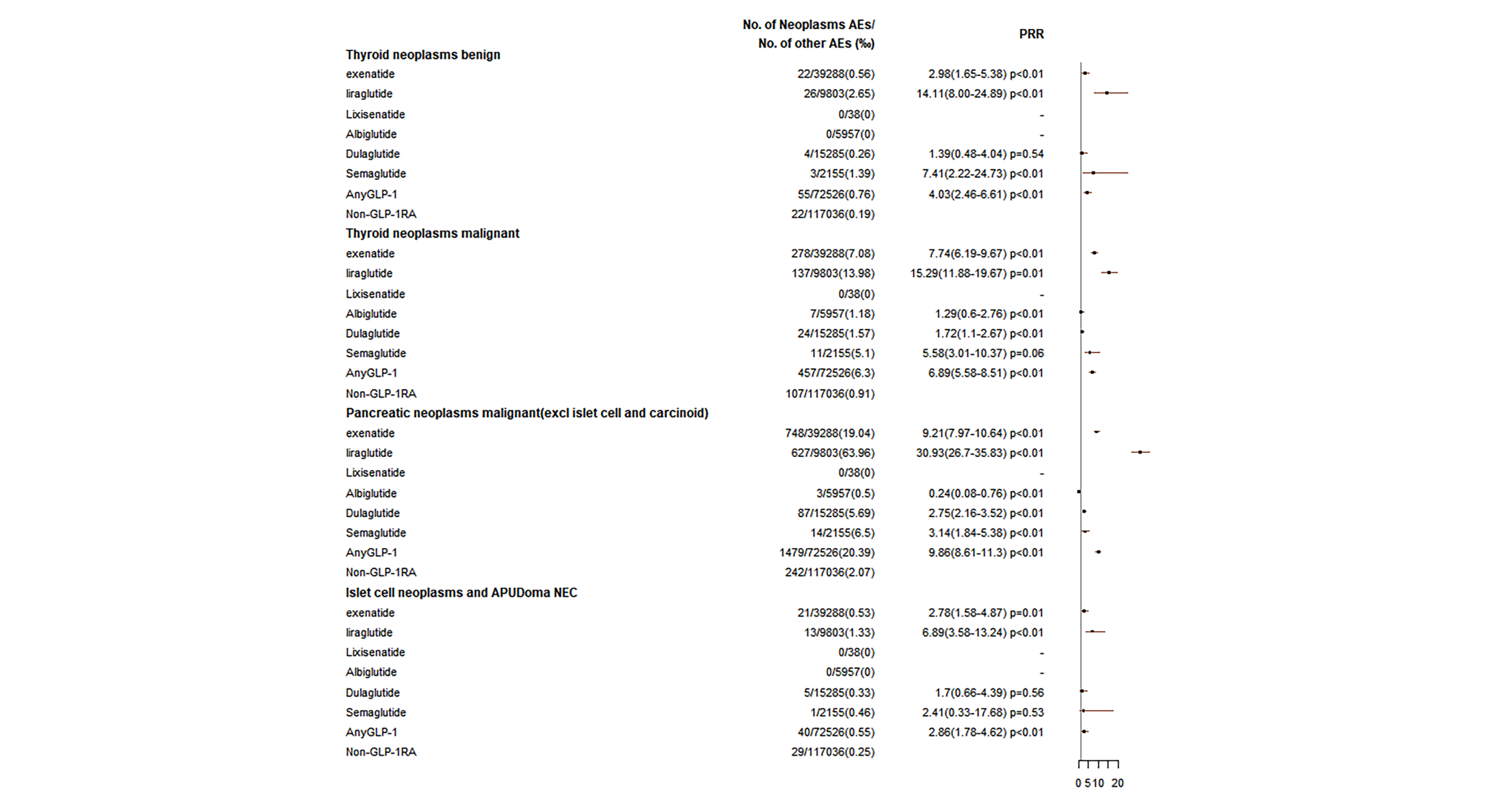


The PRR for different types of GLP-1RA-associated with thyroid neoplasms benign and malignant, pancreatic neoplasms malignant (excl islet cell and carcinoid), and ICN&AN in model 5 during 2004Q1-2021Q3.
